# Spectrophotometric Evaluation of Laser-Assisted Dental Bleaching Using Erbium-Doped Yttrium Aluminum Garnet (Er:YAG) and Diode Lasers at Different Wavelengths: An In Vitro Study

**DOI:** 10.7759/cureus.110064

**Published:** 2026-06-01

**Authors:** Esraa Ihssan Alshibli, Omar H. Hamadah, Mohammad Y. Hajeer

**Affiliations:** 1 Department of Oral Medicine, Faculty of Dentistry, Damascus University, Damascus, SYR; 2 Department of Orthodontics, Faculty of Dentistry, Damascus University, Damascus, SYR

**Keywords:** dental bleaching, diode laser, er:yag laser, hydrogen peroxide, spectrophotometry, whiteness index

## Abstract

Background

Dental bleaching is the most common method for managing tooth discoloration, and several light sources have been investigated for accelerating the bleaching process. However, various wavelengths of lasers revealed high effectiveness in tooth color whitening compared to conventional bleaching. This in-vitro study aimed to investigate the effectiveness of erbium-doped yttrium aluminum garnet (Er:YAG) and diode lasers (450 nm, 940 nm, 980 nm) in assisting in-office dental bleaching with hydrogen peroxide (HP), by evaluating tooth whitening according to the Whiteness Index (∆WIᴅ).

Materials and methods

One hundred enamel samples were obtained from 50 extracted human premolars and divided into five groups (n = 20): control with 38% HP only (first group), 38% HP + Er:YAG laser (second group), 38% HP + 450 nm diode laser (third group), 38% HP + 940 nm diode laser (fourth group), and 38% HP + 980 nm diode laser (fifth group). Tooth shade was assessed pre- and post-bleaching using a spectrophotometer. The Commission Internationale de l’Éclairage L*a*b* (CIELAB) values were obtained, and the ∆WIᴅ values were calculated for each sample. One-way ANOVA test was used to detect significant differences in ∆WIᴅ values among groups, followed by Bonferroni's post-hoc for pairwise comparisons.

Results

All tested bleaching systems were effective, showing statistically significant differences between groups (p < 0.001). The 940 nm diode laser and Er:YAG laser groups demonstrated superior whitening efficacy (18.13 ± 4.57 and 14.76 ± 6.11, respectively), followed by the 450 nm diode laser group (8.56 ± 7.56), the 980 nm diode laser group (6.74 ± 6.48), and then the control group (4.25 ± 4.67).

Conclusion

We conclude that laser-assisted dental bleaching significantly enhanced the whitening effect of HP through accelerating the bleaching process with laser energy, with the 940 nm diode laser and Er:YAG laser showing particularly promising results. This can lead to whiter teeth in fewer sessions compared to conventional bleaching.

## Introduction

Patients increasingly seek esthetic dental treatments, desiring aligned teeth with harmonious shapes, proper smile lines, and lighter shades [1]. Dental bleaching is a minimally invasive method for managing tooth discoloration [2], primarily using hydrogen peroxide (HP) or carbamide peroxide (CP) [3]. Vital tooth bleaching can be categorized as in-office bleaching, performed by professionals using high-concentration HP (30-40%), or at-home bleaching, involving patient-applied CP (10-16%) under professional supervision [4].

The bleaching mechanism relies on an oxidation process. The low molecular weight of HP enables its diffusion through enamel interprismatic spaces and dentinal tubules. It then dissociates into reactive oxygen species (ROS), which alter the chemical structure of organic pigments by converting double bonds in chromophores to single bonds. This results in lighter, lower molecular weight compounds [5,6]. This chemical reaction can be accelerated by heat or light. Various light sources have been used, including halogen lamps, plasma arc lamps, light-emitting diodes (LEDs), and lasers [7]. Laser-assisted bleaching was first introduced in 1996, and several types, including argon, potassium titanyl phosphate (KTP), neodymium-doped yttrium aluminum garnet (Nd:YAG), erbium-doped yttrium aluminum garnet (Er:YAG), and diode lasers, have since been investigated with varying outcomes [8]. Er:YAG (with a wavelength of 2940 nm) and diode lasers (with 450 nm, 810 nm, 940 nm, and 980 nm wavelengths) are considered particularly suitable for accelerating the bleaching process due to their wavelength characteristics [9]. Er:YAG laser has a high affinity for hydroxyapatite and water, and although diode lasers are highly absorbed by hemoglobin and melanin, they have demonstrated high clinical effectiveness in accelerating tooth bleaching. So, laser photonic energy is absorbed by water in the bleaching gel or by specific chromophores such as TiO_2_, generating thermal energy that promotes HP dissociation [10,11]. This can lead to satisfactory color change with reduced chair time and potentially fewer side effects [12]. Evaluating bleaching effectiveness requires accurate pre- and post-operative color measurement [13]. While visual shade matching is common, instrumental methods like spectrophotometry are preferred to avoid subjectivity [14]. A spectrophotometer provides the Commission Internationale de l’Éclairage L*a*b* (CIELAB) values, where L* represents lightness, a* represents the red-green axis, and b* represents the yellow-blue axis [15,16]. The color difference (∆E) has been widely used, but the whiteness Index (∆WIᴅ) for dentistry (WIᴅ) has shown a strong correlation with visual perception and is increasingly adopted [17].

This in-vitro study aimed to investigate the effectiveness of laser-assisted dental bleaching using Er:YAG and diode lasers (450 nm, 940 nm, 980 nm) compared to conventional bleaching, based on the WIᴅ. The null hypothesis was that all bleaching systems would produce a similar whitening effect with no significant differences.

## Materials and methods

Sample size calculation

Sample size was determined based on previous similar in-vitro studies, in which the median sample size was 15 enamel samples in each experimental group. To avoid the possibility of sample damage during the procedure, it was decided that the sample size would be 20 enamel samples in each group, so the total sample size is 100 enamel samples yielded from 50 premolars.

Sample collection and preparation

Fifty intact human premolars, extracted for orthodontic reasons, were selected. Inclusion criteria required crowns free of caries, white spot lesions, restorations, developmental defects, cracks, or fractures, as confirmed under stereoscopic microscopy (Meiji Techno Co., Ltd., Saitama, Japan) at 2x magnification. Teeth were disinfected in a chloramine solution for one week and subsequently stored in distilled water at room temperature, with weekly water changes.

**Figure 1:**
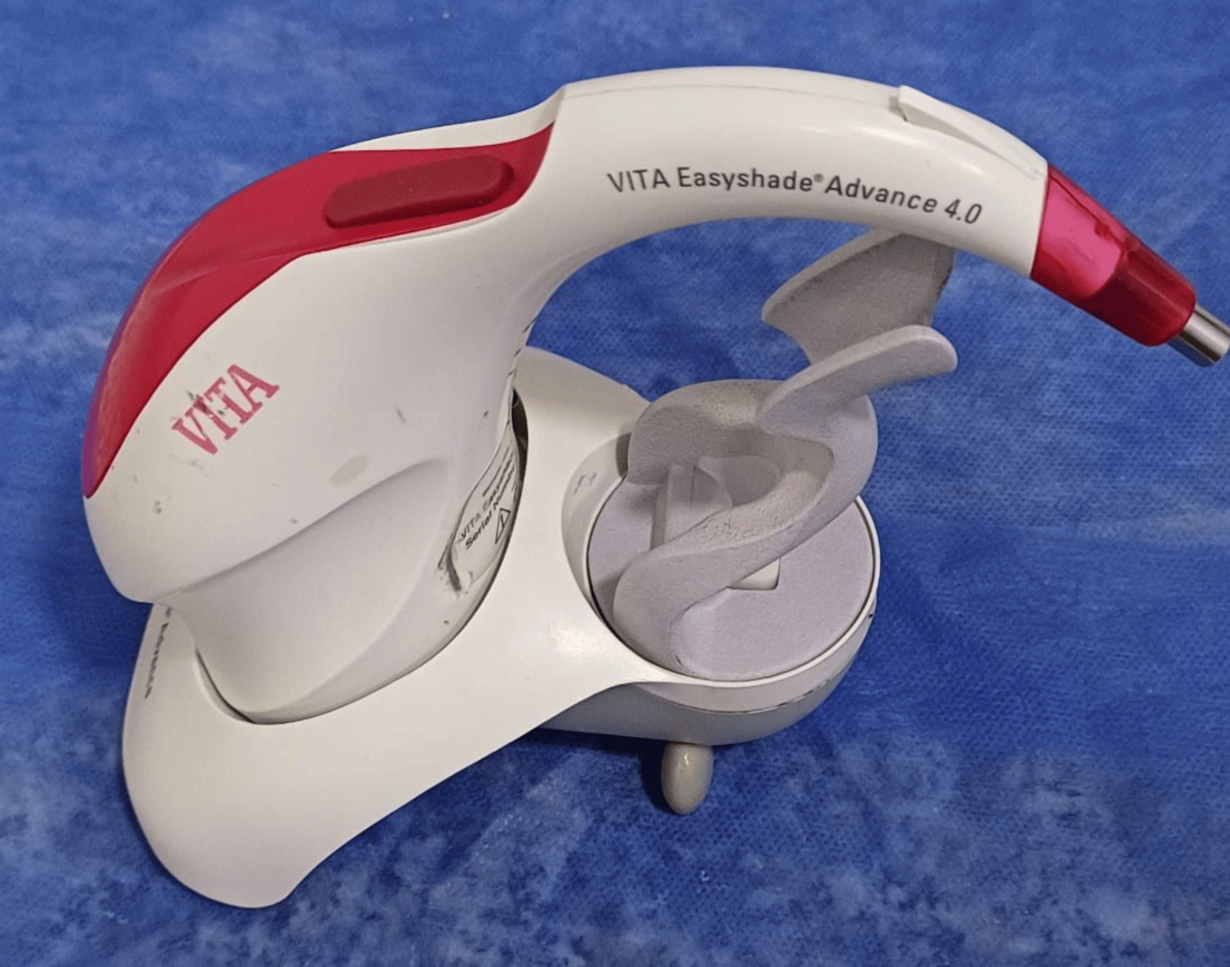
VITA Easyshade Advance 4.0 spectrophotometer (VITA Zahnfabrik H. Rauter GmbH & Co. KG, Bad Säckingen, Germany).

Each tooth was sectioned 1 mm below the cementoenamel junction and then mesiodistally into buccal and lingual halves using a low-speed handpiece with a diamond disc, yielding 100 enamel samples. Samples were embedded in acrylic resin for stable handling. Baseline color was measured using a spectrophotometer (VITA Easyshade Advance 4.0; VITA Zahnfabrik H. Rauter GmbH & Co. KG, Bad Säckingen, Germany). The device used is shown in Figure 1.

Experimental groups

Samples were randomly allocated into five groups (n=20) using an online randomizer (http://www.randomizer.org):

Group 1 (Control)

A 1-mm layer of 38% HP (mixed 30%) gel (BMS White; BMS Dental, Caputh, Germany) was applied for 15 minutes, removed via high-power suction, reapplied for another 15 minutes, and then rinsed with distilled water.

Group 2 (HP + Er:YAG)

HP gel was applied for 5 minutes, then activated with a 2940 nm Er:YAG laser (LightWalker; Fotona d.o.o., Ljubljana, Slovenia). Parameters were set at 0.5 W average power, 50 mJ/pulse, 10 Hz, VLP mode, R09 handpiece at 2 cm distance, sweeping motion for 20 seconds. This was repeated three times (60 s total irradiation). The gel remained for an additional 9 minutes before removal. The entire procedure was repeated once.

Group 3 (HP + 450 nm)

HP gel was applied for 5 minutes, then activated with a 450 nm diode laser (LX 16 Plus; Guilin Woodpecker Medical Instrument Co., Ltd., Guilin, China). Parameters were set at 1.5 W, continuous wave, bleaching handpiece at 2 cm distance for 30 seconds. This was repeated three times (90 s total) with 1-minute intervals. The gel remained for 6 minutes before removal. The procedure was repeated.

Group 4 (HP + 940 nm)

HP gel was applied for 5 minutes, then activated with a 940 nm diode laser (BIOLASE, Inc., Foothill Ranch, CA, USA). Parameters were set at 7 W, continuous wave, bleaching handpiece at 2 mm distance for 30 seconds. This was repeated twice (60 s total) with a 1.5-minute interval. The gel remained for 8 minutes before removal. The procedure was repeated.

Group 5 (HP + 980 nm)

HP gel was applied for 5 minutes, then activated with a 980 nm diode laser (Pioon Medical Laser Co., Ltd., Wuhan, China). Parameters were set at 5 W, continuous wave, bleaching handpiece at 2 cm distance for 150 seconds continuously. The gel remained for 8 minutes before removal. The procedure was repeated.

Outcome assessment

**Figure 2:**
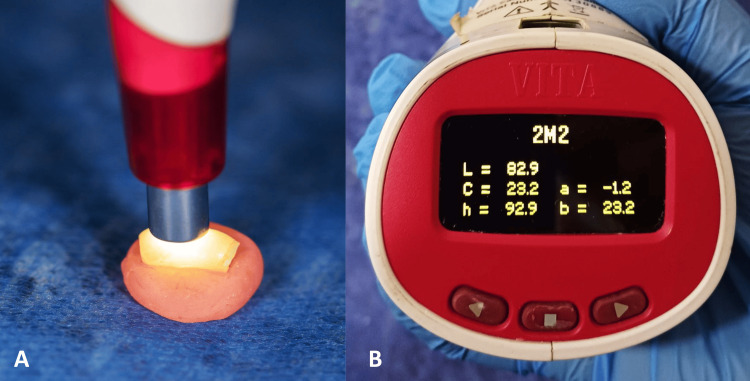
Evaluation of tooth color. A: Position the head of the device at the center of the tooth sample; B: Values expressed using the Commission Internationale de l’Éclairage L*a*b* (CIELAB) color system.

Post-bleaching color assessment was performed 24 hours later under consistent daylight conditions. The spectrophotometer tip was centrally positioned on each sample, three measurements were taken per sample, and the mean was calculated (Figure 2).

The WIᴅ was computed using the formula [18]:

$WI_D = 0.511L^{*} - 2.324a^{*} - 1.100b^{*}$

$\Delta WI_D = WI_D(\mathrm{post-bleaching}) - WI_D(\mathrm{baseline})$

Statistical analysis

Data were analyzed using IBM SPSS Statistics for Windows, Version 25 (Released 2017; IBM Corp., Armonk, New York, United States). Normality was assessed with the Shapiro-Wilk test. One-way ANOVA compared the ∆WIᴅ between groups, followed by Bonferroni's post-hoc test for pairwise comparisons. Significance was set at p < 0.05.

## Results

The ∆WIᴅ mean values and standard deviations are presented in Table 1. One-way ANOVA confirmed that all systems were effective, with significant differences between groups (p < 0.001).

**Table 1:** Descriptive statistics and significant differences in mean Whiteness Index values (∆WIᴅ) among the study groups. ^†^ Employing one-way ANOVA; F-value (95.4) = 18.484. ANOVA: analysis of variance; SD: standard deviation; Er:YAG: erbium-doped yttrium aluminum garnet

Study Group	Mean ∆WIᴅ ± SD	p-value^†^
Group 1 (control)	4.25 ± 4.67	<0.001
Group 2 (Er:YAG)	14.76 ± 6.11	
Group 3 (450 nm diode)	8.56 ± 7.56	
Group 4 (940 nm diode)	18.13 ± 4.57	
Group 5 (980 nm diode)	6.74 ± 6.48	

Post-hoc Bonferroni tests (Table 2) revealed significant differences between the control group and both the Er:YAG and 940 nm groups (p < 0.001), and significant differences were also observed between the Er:YAG group and both the 450 nm (p = 0.015) and 980 nm (p = 0.001) groups. In addition, a significant difference was found between the 940 nm group and both the 450 nm and 980 nm groups (p < 0.001). No significant difference was found between the control group and the 450 nm or 980 nm groups, or between the Er:YAG and 940 nm groups.

**Table 2:** Post hoc pairwise comparisons of whiteness index (ΔWIᴅ) values among the study groups. ^†^ Employing Bonferroni's post hoc tests Er:YAG: erbium-doped yttrium aluminum garnet

Compared Groups	Mean Difference	p-value^†^
Control vs. Er:YAG	-10.52	<0.001
Control vs. 940 nm diode	-13.89	<0.001
Er:YAG vs. 450 nm diode	6.20	0.015
Er:YAG vs. 980 nm diode	8.02	0.001
940 nm diode vs. 450 nm diode	-9.58	<0.001
940 nm diode vs. 980 nm diode	-11.39	<0.001

## Discussion

This study evaluated the effect of laser-assisted bleaching using Er:YAG and diode lasers at different wavelengths. To our knowledge, it is the first in-vitro study to employ the WIᴅ for this purpose. This study was carried out on human premolars because of the frequency of their extraction for orthodontic reasons, allowing for their acquisition in a sound condition according to the inclusion criteria. For the color assessment, all measurements were performed under daylight in uniform conditions using VITA Easyshade to avoid subjectivity and obtain measurable data represented by CIELAB values. The spectrophotometer tip was centrally positioned on each sample to ensure reproducibility, as the middle third of the tooth is the region that best represents tooth color compared to the gingival and incisal thirds, due to the variation in enamel thickness in those areas [19].

The demand for effective bleaching has driven research into various agents and activation methods [20]. The WIᴅ, which correlates well with visual perception, provides a clinically relevant measure of whitening, reflecting increases in lightness and reductions in chroma [14,21]. Although previous studies used other indices, such as ∆Eaь and ∆Eₒₒ, for evaluating whiteness after bleaching procedures [22-24], a sole evaluation of color differences was not adequate and did not offer enough information on how color coordinates change [25]. However, WIᴅ was specifically designed for dentistry, can be easily implemented in instrumental color measurements [25,26], and has a clear and simple interpretation where high values of ∆WIᴅ indicate higher whiteness, while low or even negative values indicate lower whiteness [27]. Therefore, this index was taken into consideration in this study.

Laser application is effective in dental bleaching as it accelerates the chemical reaction by converting laser irradiation into heat, which is absorbed by the whitening gel [28]; this effect depends on the laser wavelength and the composition of the bleaching agent used [29,30].

The results demonstrate that all laser-assisted protocols were effective (∆WIᴅ > 2.6, the threshold for clinical perceptibility) [17], leading to the rejection of the null hypothesis. The 940 nm diode laser group achieved the highest ∆WIᴅ, likely due to its strong absorption by pigments within the bleaching gel, thereby accelerating radical formation [31]. The Er:YAG laser also showed high efficacy, attributable to its affinity for water and hydroxyapatite, which constitutes a significant portion of the gel, enhancing HP dissociation [8,32].

The 450 nm and 980 nm diode lasers improved whitening compared to the control, albeit to a lesser extent, supporting the general photothermal benefit of laser activation [33-35]. These findings align with a previous in-vitro study on bovine enamel, which reported superior ∆WIᴅ with a hybrid light system activating TiO_2_-doped bleaching gel [36]. Only one in-vivo study investigated the effect of 808 nm diode laser on 35% HP efficacy based on the ∆WIᴅ. The results revealed that a significant whitening was observed after bleaching for both conventional and laser-assisted dental bleaching, with no significant difference between groups [37].

Limitations of the current work

A primary limitation of this study is its in-vitro design, which cannot fully replicate the complexity of the oral environment. Future in vivo studies with different bleaching agent concentrations, laser parameters, and long-term follow-up to assess color stability are warranted.

## Conclusions

Within the limitations of this study, and based on ∆WIᴅ, we can conclude that dental bleaching with 38% HP enhanced tooth whitening, whether conventional or activated. Laser-assisted dental bleaching with Er:YAG and diode lasers at the tested wavelengths significantly enhanced the bleaching effect of 38% HP. The 940 nm diode laser performed best in this study, and the Er:YAG laser demonstrated superior effectiveness compared to the other diode wavelengths and conventional bleaching. So, laser-assisted dental bleaching with Er:YAG and (450 nm, 940 nm, 980 nm) diode lasers provides an effective method to gain whiter teeth in a few sessions.
